# Efficacy of hypothermic oxygenated machine perfusion in liver transplantation: a trial sequential analysis

**DOI:** 10.1097/JS9.0000000000001338

**Published:** 2024-03-18

**Authors:** I-Wen Chen, Wei-Ting Wang, Shu-Wei Liao, Kuo-Chuan Hung

**Affiliations:** a Department of Anesthesiology, Chi Mei Medical Center, Liouying; b Department of Anesthesiology, Chi Mei Medical Center, Tainan City; c Department of Anesthesiology, E-Da Hospital, I-Shou University, Kaohsiung City, Taiwan

*Dear Editor,*

We read with great interest the article ‘Hypothermic oxygenated perfusion in liver transplantation: a meta-analysis of randomized controlled trials and matched studies’ by Tang *et al*.^1^ recently published in the *International Journal of Surgery*. The authors performed a comprehensive meta-analysis evaluating the efficacy of hypothermic oxygenated machine perfusion (HOPE) compared with static cold storage (SCS) in liver transplantation. The key findings were that HOPE effectively reduced biliary complications, non-anastomotic biliary strictures, early allograft dysfunction, acute rejection, retransplantation rates, and 1-year graft loss compared with SCS. This suggests that HOPE protects against ischemia-reperfusion injury. Their findings are important, as HOPE is a novel organ preservation technique that may help expand the donor pool and improve outcomes of liver transplantation. We congratulate Tang *et al*.^1^ for conducting this timely and informative meta-analysis. These results offer evidence to support the wider adoption of HOPE in liver transplant programs globally.

The authors reported that HOPE significantly reduced early allograft dysfunction by 46% [risk ratio (RR) 0.54] and 1-year graft loss rates by 62% (RR 0.38) compared with SCS^1^. These are critically important outcomes in liver transplantation that affect short-term and long-term patient and graft survival. However, the reliability of these results needs to be assessed before changing the clinical practice based on this evidence. One method to evaluate the robustness of cumulative data in a meta-analysis is trial sequential analysis (TSA)^2–4^. TSA calculates the information size (i.e. number of participants) required to demonstrate a statistically significant intervention effect. It then constructs boundaries to determine whether the evidence is conclusive or if additional studies are needed to reach the optimal information size and firm conclusion^2^. If the cumulative *z*-curve crosses the trial sequential monitoring boundary or the required information size boundary, robust evidence for efficacy exists. Therefore, TSA provides invaluable insights into whether the available data are adequately powered for clinical decision-making.

To assess the robustness of the evidence for the key outcomes of early allograft dysfunction and 1-year graft loss, we performed a TSA using the raw data from the original meta-analysis^1^. For early allograft dysfunction, TSA demonstrated that the cumulative *z*-curve crossed the TSA boundary (Fig. 1A), establishing sufficient evidence to show a 46% risk reduction with HOPE (RR, 0.54). This indicates that the current pooled data on early allograft dysfunction are conclusive, and HOPE should be recommended to reduce early allograft dysfunction. However, for 1-year graft loss, the *z*-curve did not cross the trial sequential monitoring boundary (Fig. 1B). Despite a 62% risk reduction (RR 0.38), more evidence is required to reach firm conclusions about this long-term outcome.

**Figure 1:**
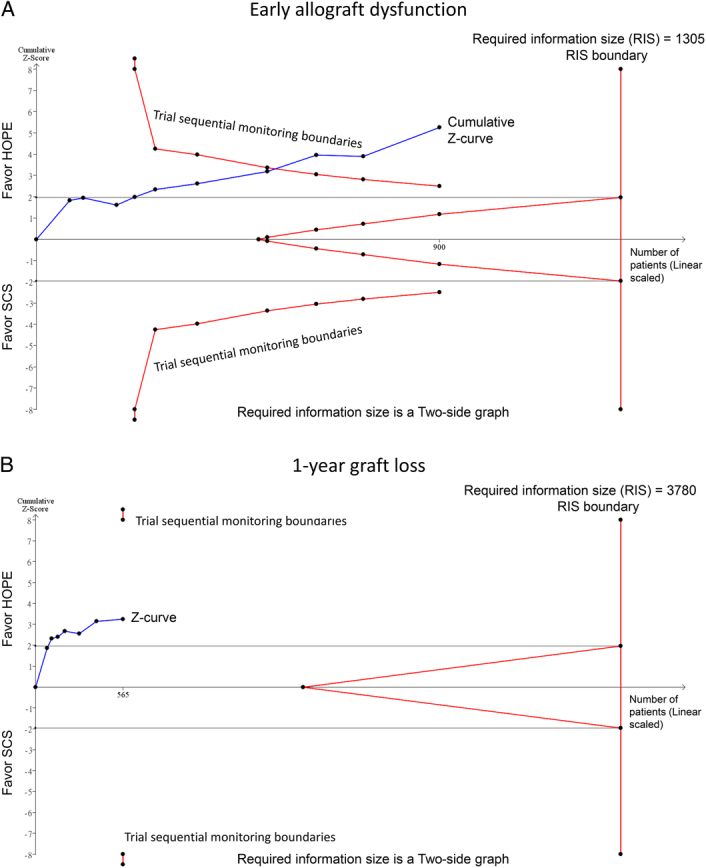
Displays the results of a trial sequential analysis (TSA) on the efficacy of hypothermic oxygenated machine perfusion (HOPE) compared with static cold storage (SCS) in liver transplantation, based on data from Tang *et al*.’s meta-analysis. (A) For early allograft dysfunction, the cumulative *z*-curve (blue line) crosses the trial sequential monitoring boundary (red lines), indicating that the pooled data provide sufficient evidence to demonstrate a 46% reduction in the risk of early allograft dysfunction with HOPE treatment (RR, 0.54). (B) For the outcome of 1-year graft loss, the *z*-curve does not cross the trial sequential monitoring boundary, suggesting that, despite a 62% reduction in risk (RR, 0.38), the evidence is not yet conclusive.

In conclusion, cumulative evidence suggests that HOPE has great potential to reduce early allograft dysfunction. The results on 1-year graft loss are very encouraging but warrant caution as TSA shows that more data is still needed to conclusively recommend adopting HOPE solely to reduce graft loss. Continued, adequately powered, high-quality studies are necessary to reach the optimal information size and provide confirmatory evidence of the long-term impact.

## Ethical approval

Not applicable.

## Consent

Not applicable.

## Sources of funding

Not applicable.

## Author contribution

I-W.C. and K.-C.H.: wrote the main manuscript text; W.-T.W. and S.-W.L.: prepared Figure 1. All authors read and approved the final version of the manuscript.

## Conflicts of interest disclosure

The authors declare no conflicts of interest.

## Research registration unique identifying number (UIN)

Not applicable.

## Guarantor

Kuo-Chuan Hung.

## Data availability statement

The datasets used and/or analyzed in the current study are available from the corresponding author upon reasonable request.

## Provenance and peer review

This paper was not invited.
